# Occupational radiation exposure and glaucoma and macular degeneration in the US radiologic technologists

**DOI:** 10.1038/s41598-018-28620-6

**Published:** 2018-07-11

**Authors:** Mark P. Little, Cari M. Kitahara, Elizabeth K. Cahoon, Marie-Odile Bernier, Raquel Velazquez-Kronen, Michele M. Doody, David Borrego, Jeremy S. Miller, Bruce H. Alexander, Steven L. Simon, Dale L. Preston, Craig Meyer, Martha S. Linet, Nobuyuki Hamada

**Affiliations:** 1 Radiation Epidemiology Branch, Division of Cancer Epidemiology and Genetics, National Cancer Institute, National Institutes of Health, Department of Health and Human Services, Bethesda, Maryland 20892-9778 USA; 2 Laboratory of Epidemiology, Institut de Radioprotection et de Sûreté Nucléaire, Fontenay aux Roses, France; 3 Information Management Services, Silver Spring, Maryland 20904 USA; 4 Division of Environmental Health Sciences, School of Public Health, University of Minnesota, Minneapolis, Minnesota 55409 USA; 5 Hirosoft International, Eureka, California, 95501 USA; 6 Radiation Safety Research Center, Nuclear Technology Research Laboratory, Central Research Institute of Electric Power Industry (CRIEPI), 2-11-1 Iwado-kita, Komae, Tokyo 201-8511 Japan

## Abstract

There are well-documented associations of glaucoma with high-dose radiation exposure, but only a single study suggesting risk of glaucoma, and less conclusively macular degeneration, associated with moderate-dose exposure. We assessed risk of glaucoma and macular degeneration associated with occupational eye-lens radiation dose, using participants from the US Radiologic Technologists Study, followed from the date of surveys in 1994–1998, 2003–2005 to the earliest of diagnosis of glaucoma or macular degeneration, cancer other than non-melanoma skin cancer, or date of last survey (2012–2014). We excluded those with baseline disease or previous radiotherapy history. Cox proportional hazards models with age as timescale were used. There were 1631 cases of newly self-reported doctor-diagnosed cases of glaucoma and 1331 of macular degeneration among 69,568 and 69,969 eligible subjects, respectively. Estimated mean cumulative eye-lens absorbed dose from occupational radiation exposures was 0.058 Gy. The excess relative risk/Gy for glaucoma was −0.57 (95% CI −1.46, 0.60, *p* = 0.304) and for macular degeneration was 0.32 (95% CI −0.32, 1.27, *p* = 0.381), suggesting that there is no appreciable risk for either endpoint associated with low-dose and low dose-rate radiation exposure. Since this is the first examination of glaucoma and macular degeneration associated with low-dose radiation exposure, this result needs to be replicated in other low-dose studies.

## Introduction

In 2013, the total economic burden of vision loss and blindness in the US was estimated to be $139 billion, and treatment of eye-related disorders totalled more than $68.8 billion in annual direct medical costs^1^. Among the largest component of costs are those due to cataract, macular degeneration and glaucoma^1^. Macular degeneration, which is comprised of two major subtypes, “wet” and “dry” disease^2^, is the leading cause of vision loss in the US and in other developed countries^3–5^, but worldwide glaucoma, the main four subtypes of which are primary open-angle glaucoma (the most common type in the US), primary angle-closure glaucoma, secondary glaucoma, and congenital glaucoma^6^, has greater impact on vision loss, second only to cataract^7^.

Diabetes, obesity, and other types of metabolic disease are associated with some types of glaucoma^8,9^. Age, cigarette smoking and genetic risk are the well-established major environmental and lifestyle risk factors associated with macular degeneration^10^; there is some evidence also for risks associated with obesity, certain nutritional components (in particular increased total fat) and other factors linked with circulatory disease^11–14^. Solar exposure has been found to be correlated with macular degeneration, but the direction of the association is in doubt^15,16^; there is little data, even of this contradictory sort, suggesting a link of solar exposure with glaucoma.

There are well-documented associations of (often neovascular) glaucoma with high dose radiotherapeutic procedures^17,18^. Only normal-tension glaucoma has been observed in excess in the Japanese atomic-bomb survivors^19^. There is evidence of association of retinal degeneration with radiation dose in the Japanese atomic-bomb survivors^20^, but no association with macular degeneration as such^21^. There is no other data at moderate or low doses for either endpoint. The National Council on Radiation Protection and Measurements recommended that parts of the eye other than the eye lens need to be taken into account in the system of radiological protection^22^.

In this report, we analysed glaucoma and macular degeneration in the US Radiologic Technologist (USRT) cohort in relation to cumulative absorbed dose from occupational radiation exposures. The analysis used recently updated and improved eye-lens dosimetry^23^. The analysis is the first such to address these ocular endpoints in a large cohort exposed predominantly to low doses and low dose-rates of ionising radiation.

## Results

There were 1631 cases of glaucoma with 921,076 years of follow-up among 69,568 subjects eligible for the glaucoma analysis, and 1331 cases of macular degeneration with 930,098 person years of follow-up among 69,969 subjects eligible for the macular degeneration analysis (Table 1), implying 13.2 and 13.3 years of follow per person for glaucoma and macular degeneration, respectively. The mean age at entry and exit for persons eligible to study glaucoma were 48.1 (interquartile range 41.5–52.9) and 61.3 (interquartile range 55.6–66.5) years, while for macular degeneration the analogous figures were 48.1 (interquartile range 41.5–53.0) and 61.4 (interquartile range 55.6–66.6) years (data not shown). The mean age at diagnosis of glaucoma and macular degeneration cases was 60.0 (interquartile range 53.5–66.5) and 64.7 (interquartile range 57.5–72.5) (data not shown). The cumulative mean eye-lens absorbed dose was 0.058 Gy (interquartile range 0.024–0.071 Gy) in the glaucoma study group and in the macular degeneration study group. Glaucoma risk was elevated in those with diabetes at baseline (heterogeneity-*p* < 0.001), and among blacks and other races compared to whites (heterogeneity-*p* < 0.001). There were also indications of elevated risk among the obese (BMI ≥ 30 kg/m^2^) and among heavy smokers (≥40 cigarettes/day). Macular degeneration risk was likewise increased in diabetics (heterogeneity-*p* < 0.001), but in contrast to glaucoma, blacks and other races were at significantly lower risk than whites (heterogeneity-*p* < 0.001). Macular degeneration risks were also elevated among the obese (heterogeneity-*p* < 0.001), among cigarette smokers (heterogeneity-*p* = 0.002), in particular, among heavy cigarette smokers (≥40 cigarettes/day) (heterogeneity-*p* = 0.007), and among those who stopped smoking at later ages (heterogeneity-*p* < 0.001) (Table 1).

**Table 1:** Numbers of cases and non-cases of glaucoma and macular degeneration in US radiologic technologists in relation to various risk factors, person years of follow-up and relative risks (hazard ratios) (evaluated via Cox regression) (+95% CI).

	Glaucoma					Macular degeneration				
	Cases	Non-cases	Person years	Relative risk	*p*-value	Cases	Non-cases	Person years	Relative risk	*p*-value
TOTAL	1631	67,937	921,076			1331	68,638	930,098		
**Sex**										
Male	389	14,687	194,670	1 (reference)	0.531	340	14,868	196,997	1 (reference)	0.632
Female	1,242	53,250	726,406	0.96 (0.86, 1.08)		991	53,770	733,101	0.97 (0.86, 1.10)	
**Racial/ethnic group**										
White	1,483	64,569	877,062	1 (reference)	<0.001	1,282	65,104	884,626	1 (reference)	<0.001
Black	100	1,812	22,856	2.23 (1.82, 2.73)		22	1,937	23,943	0.48 (0.31, 0.73)	
Other	48	1,556	21,158	1.26 (0.94, 1.67)		27	1,597	21,529	0.77 (0.53, 1.13)	
**Diabetes at baseline**										
None	1,462	63,795	875,139	1 (reference)	<0.001	1,214	64,365	882,591	1 (reference)	<0.001
Missing	76	2,585	27,112	1.39 (1.10, 1.75)		47	2,624	27,560	0.91 (0.68, 1.21)	
Diabetes	93	1,557	18,825	2.30 (1.86, 2.84)		70	1,649	19,947	1.73 (1.36, 2.21)	
**Body mass index (BMI) (kg/m** ^**2**^ **) at baseline**										
18.5–24.9 (normal)	741	33,689	463,628	1 (reference)	0.127	578	33,969	467,106	1 (reference)	0.001
Missing	35	1,551	19,457	1.03 (0.73, 1.44)		19	1,583	19,946	0.67 (0.42, 1.06)	
0–18.4	15	928	12,244	0.81 (0.48, 1.35)		15	930	12,335	0.99 (0.59, 1.65)	
25.0–29.9	539	20,795	280,014	1.06 (0.95, 1.19)		464	21,024	282,957	1.10 (0.98, 1.25)	
≥30.0	301	10,974	145,733	1.18 (1.04, 1.35)		255	11,132	147,753	1.31 (1.13, 1.52)	
**Baseline smoking status**										
Never smoked	854	38,150	523,637	1 (reference)	0.371	648	38,541	527,987	1 (reference)	0.002
Missing smoking status	11	567	7,183	0.75 (0.41, 1.36)		11	570	7,292	0.76 (0.42, 1.38)	
Former smoker	562	20,731	279,892	1.06 (0.95, 1.18)		495	20,961	283,266	1.15 (1.02, 1.29)	
Current smoker	204	8,489	110,364	1.09 (0.94, 1.27)		177	8,566	111,553	1.34 (1.14, 1.59)	
**Baseline smoking quantity (cigarettes/day)**										
Missing/never smoker	898	39,698	547,047	1 (reference)	0.179	686	40,094	551,669	1 (reference)	0.007
0–9	151	5,358	70,899	1.19 (1.00, 1.41)		108	5,445	71,767	1.06 (0.87, 1.30)	
10–19	234	9,772	130,251	1.02 (0.88, 1.18)		204	9,844	131,471	1.15 (0.99, 1.35)	
20–29	236	9,490	125,754	1.01 (0.88, 1.17)		230	9,579	127,232	1.27 (1.09, 1.47)	
30–39	59	2,214	28,899	1.05 (0.81, 1.37)		55	2,247	29,336	1.25 (0.95, 1.65)	
≥40	53	1,405	18,226	1.37 (1.03, 1.80)		48	1,429	18,623	1.49 (1.11, 1.99)	
**Baseline age at stopping smoking (years)**										
Never stopped/never smoked	901	39,848	549,380	1 (reference)	0.209	677	40,249	553,898	1 (reference)	<0.001
0–19	10	407	5,642	1.24 (0.67, 2.32)		7	408	5,652	1.38 (0.66, 2.91)	
20–29	152	7,049	97,446	0.98 (0.82, 1.16)		109	7,127	98,369	1.03 (0.84, 1.26)	
30–39	167	7,856	107,045	0.97 (0.82, 1.14)		116	7,975	108,523	0.92 (0.75, 1.12)	
40–49	209	8,170	107,900	1.10 (0.95, 1.28)		170	8,271	109,093	1.26 (1.07, 1.49)	
50–59	153	3,635	43,906	1.25 (1.05, 1.49)		175	3,672	44,838	1.63 (1.38, 1.93)	
≥60	39	972	9,757	1.10 (0.79, 1.53)		77	936	9,725	1.54 (1.21, 1.97)	

Relative risks were derived using univariate Cox proportional hazards models, with age as timescale. *p*-values of heterogeneity are given.

Table 2 demonstrates that there were weak indications of increased risk for both endpoints at low eye-lens absorbed doses, up to about 0.02 Gy, although the excess risk for both endpoints largely disappeared at doses above 0.1 Gy.

**Table 2:** Numbers of cases of glaucoma, and macular degeneration in US radiologic technologists in relation to cumulative eye-lens absorbed dose from occupational exposures, person years of follow-up and unadjusted relative risks (hazard ratios) (via Cox regression using age as timescale) (+95% CI).

Eye-lens absorbed dose (Gy)	Glaucoma					Macular degeneration				
	Cases	Non-Cases	Person Years	Relative risk	*p*-value	Cases	Non-Cases	Person Years	Relative risk	*p*-value
<0.005	30	1202	15,304	1 (reference)	0.005	18	1215	15,499	1 (reference)	0.026
0.005–0.009	40	2259	30,636	1.25 (0.78, 2.01)		22	2286	30,906	1.25 (0.83, 1.89)	
0.010–0.019	143	9314	130,407	1.14 (0.77, 1.70)		97	9392	131,132	1.19 (0.84, 1.68)	
0.020–0.049	550	27,772	386,639	1.06 (0.74, 1.54)		369	28,068	389,992	1.12 (0.81, 1.55)	
0.050–0.099	532	18,144	246,211	0.99 (0.68, 1.43)		389	18,415	249,425	1.02 (0.74, 1.41)	
0.10–0.19	268	7314	91,842	0.84 (0.58, 1.23)		279	7399	93,202	0.97 (0.69, 1.34)	
0.20–0.49	65	1797	18,995	0.70 (0.45, 1.08)		134	1752	18,982	0.85 (0.58, 1.23)	
≥0.50	3	135	1,042	0.51 (0.15, 1.70)		23	111	958	0.47 (0.17, 1.35)	

Unadjusted relative risks were derived using a Cox proportional hazards model in relation to (non-time varying) eye lens dose cumulative to 1997, with age as timescale, unadjusted for any other covariate. *p*-values of heterogeneity are given.

**Figure 1:**
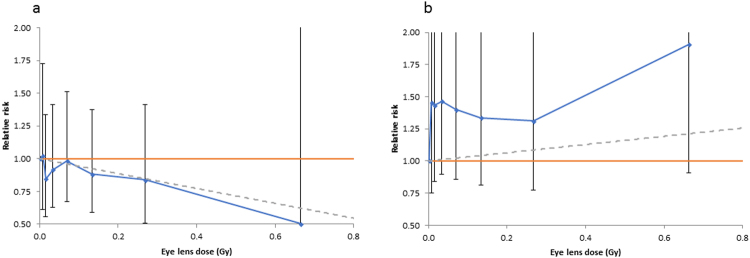
Adjusted relative risks (hazard ratios) and linear dose response estimates for (**a**) glaucoma and (**b**) macular degeneration in US radiologic technologists in relation to 5-year lagged cumulative eye-lens absorbed dose from occupational exposures (+95% CI). Risks are evaluated using a Cox model with age as timescale with stratification by sex, race and birth year (by decade <1900, 1900–1909, …, 1950–1959, ≥1960), and with adjustment to the baseline hazard for diabetes, body mass index (BMI), smoking status (current smoker/ex-smoker/never smoker, numbers of cigarettes/day, age stopped smoking), each ascertained at the baseline survey. For each endpoint (glaucoma, macular degeneration) follow-up is restricted to those persons eligible for study (no record of radiotherapy or disease at baseline, unambiguous status at end of follow-up (with both fact and year of diagnosis known) etc) (see Methods).

Table 3 (and Fig. 1) reveals that the declining risk with increasing radiation dose for glaucoma and the increasing risk with increasing radiation dose for macular degeneration in the unadjusted results became non-significant after adjustment for lifestyle and medical factors specified *a priori*. The absorbed dose excess relative risk (ERR) / Gy for glaucoma was −0.57 (95% CI −1.46, 0.60, *p* = 0.304) and for macular degeneration was 0.32 (95% CI −0.32, 1.27, p = 0.381) (Table 3). Little difference was made to the trend risk estimate or to the overall goodness of fit by variation of the lagging period between 0 and 10 years (results not shown). When persons with prior radiotherapy recorded on the first two questionnaires were added back in, or when the censoring at occurrence of cancer other than NMSC was removed, or both, little difference was made to glaucoma or macular degeneration risk (Supplementary Information Part C Table 1).

**Table 3:** Excess relative risks for glaucoma and macular degeneration in US radiologic technologists in relation to lagged cumulative eye-lens absorbed dose from occupational exposures (+95% CI).

		Unadjusted relative risks		Adjusted relative risks	
Endpoint	Cases	ERR (+95% CI)	*p*-value	ERR (+95% CI)	*p*-value
Glaucoma	1631	−1.39 (−1.94, −0.69)	<0.001	−0.57 (−1.46 ^W^, 0.60)	0.304
Macular degeneration	1331	0.73 (−0.01, 1.74)	0.052	0.32 (−0.32, 1.27)	0.381

Unadjusted relative risks were derived using a Cox proportional hazards model in relation to 5-year lagged cumulative eye lens dose, with age as timescale, unadjusted for any other covariate. For each endpoint (glaucoma, macular degeneration) follow-up is restricted to those persons eligible for study (no record of radiotherapy or disease at baseline, unambiguous status at end of follow-up (with both fact and year of diagnosis known) etc)(see Methods). Adjusted relative risks are evaluated using a Cox model with age as timescale, with stratification by sex, race and birth year (by decade <1900, 1900–1909, …, 1950–1959, ≥ 1960), and with adjustment to the baseline hazard for diabetes, body mass index (BMI), smoking status (current smoker/ex-smoker/never smoker, numbers of cigarettes/day, age stopped smoking), each ascertained at the baseline survey. For each endpoint (glaucoma, macular degeneration) follow-up is restricted to those persons eligible for study (no record of radiotherapy or disease at baseline, unambiguous status at end of follow-up (with both fact and year of diagnosis known) etc)(see Methods). *p*-values of improvement in fit over null model (with no trend in dose) are given, assessed via the likelihood ratio test. Unless otherwise indicated all confidence intervals are derived from the profile likelihood; those not so based are derived using Wald-based methods, and indicated with a superscript W.

## Discussion

We found no significant radiation-associated excess risk for glaucoma or macular degeneration in analyses adjusted for other covariates. The novelty of this large occupational study is that, in contrast to the few previous studies of glaucoma or macular degeneration, the dose rates are all low (<5 mGy/hour) and the cumulative absorbed doses are also mostly low (<0.1 Gy)^24^. The study is also unusual in that it has a rich set of individual lifestyle and environmental covariate data, some of which were used to adjust baseline risk. Risks for both endpoints were significantly associated with lifestyle and medical risk factors, in particular, diabetes, obesity, and race (Table 1). There were indications that both endpoints were elevated among smokers, the increase being particularly strong for macular degeneration.

Diabetes and obesity are well established risk factors for glaucoma^8,9^ and they were highly significantly related to the outcomes in our study, with increased risks particularly marked for those with diabetes (Table 1). Neovascular glaucoma is frequently observed after radiation treatment for uveal melanoma. Mean radiation doses to the eye from ^125^I brachytherapy used for treating uveal melanoma are typically about 75–85 Gy, delivered in a single fraction to the tumour apex, with dose rates of 0.4–1.2 Gy/h^25,26^. Use of proton beam therapy to treat uveal melanoma may deliver slightly less dose, 50–70 Gy, generally in 5 fractions^27^, similar to other types of external beam therapy^28^. The prevalence of glaucoma after ^125^I radiation treatment for uveal melanoma is very high – about 50% of patients so treated will develop glaucoma, generally of secondary and specifically neovascular type, within 3 years of treatment^29^. Prevalence of (generally neovascular) glaucoma may be slightly less, ~25%, in patients treated with a proton beam^27^, possibly because of the slightly lower dose and the dose fractionation. The prevalence of neovascular glaucoma was still lower, ~9%, after LINAC treatment^28^, based on a median follow-up of 20 months. The prevalence of neovascular glaucoma (NVG) declined dramatically from 48% (15/31) to 9% (3/33), with a relatively modest reduction in the gamma knife radiosurgery dose from 52.1 Gy to 41.5 Gy^30^. A problem with causal interpretation of this body of radiotherapy data is that larger uveal melanomas, which are more likely to in-grow, are treated with higher cumulative radiation to collateral structures that likely results in greater iris microvascular injury^31^, so that radiation dose is largely confounded with tumour size. Nevertheless, glaucoma appears to conform to what would be expected of a tissue reaction (formerly deterministic) effect^32^. The type of glaucoma associated with radiotherapy, generally neovascular glaucoma, is pathophysiologically distinct from open-angle glaucoma, which is the most common form of glaucoma and *a priori* the glaucoma subtype likely to account for most of the glaucoma cases in this cohort. It is unlikely that an appreciable proportion of the cohort would have had the large doses to the eye that would result in a neovascular glaucoma, although it is possible that some of the other well-established causes of neovascular glaucoma, for example central retinal vein occlusion and diabetic retinopathy,^33^ could apply at the generally low doses that pertain here. After high doses, neovascular glaucoma typically develops relatively quickly, within 3 years after radiotherapy^28,29^; it is possible that both it and other types of glaucoma develop more slowly after lower doses, like other tissue reaction effects^32^, although there is no evidence of that here. In contrast to the situation at radiotherapeutic levels of dose, at the much lower doses received by the atomic bomb survivors normal-tension glaucoma, a subtype of primary open-angle glaucoma, was the only glaucoma subtype associated with radiation exposure^19^. The risks factors apart from radiation were quite distinct for normal-tension glaucoma in the LSS, limited to hypertension and obesity (with increased risk for those underweight)^19^. From the fact that glaucoma risks in our study were elevated among the obese and not among the underweight (Table 1) suggests that the subtype examined here is likely mostly not normal-tension glaucoma.

Age, cigarette smoking and genetic risk factors are well-established risk factors for macular degeneration, and there is emerging evidence also for obesity, high serum cholesterol and other factors linked with circulatory disease^10–14,34^. In contrast to glaucoma, there does not seem to be, even at high dose, much evidence of radiation association with macular degeneration, although radiation retinopathy is well documented following radiotherapy of the retina and associated parts of the eye^35,36^. Radiotherapy has in fact been used to treat macular degeneration, although this has had mixed results^37^. As noted in the Methods, for this endpoint and for glaucoma diabetes is likely to result in increased frequency of eye exam, with consequent increased likelihood of detection of the disease. As such, it is important to adjust for diabetes status in the way we do (Table 3, Fig. 1), irrespective of its status as a risk factor.

A strength of this study is its large size, prospective design, and the fact that adjustment was made for several factors that have been associated with glaucoma and macular degeneration, including diabetes, obesity, and cigarette smoking. Adjustment for these variables had some effect on radiation risks, so that without adjustment for them, there was a strong and statistically significant negative dose trend for glaucoma, and a strong and borderline significant positive trend with dose for macular degeneration. These unadjusted results likely demonstrate the impact of uncontrolled confounding. A comprehensive historical dose reconstruction estimated annual and cumulative badge and organ-specific (including lens of the eye) absorbed doses for each study participant based on actual badge dose readings, detailed work history data, and extensive review of the literature and other archives^23^, as discussed in more detail in Supplementary Information Part A. The dosimetry has been subject to extensive validation, in particular, via chromosome aberrations detected using fluorescence *in situ* hybridisation (FISH)^38^.

A weakness of the study is that all work history data and clinical disease outcomes were ascertained solely by questionnaire. Since disease outcomes were not validated, the specific types of glaucoma and macular degeneration were unknown. This is a complication in making comparisons with findings in other cohorts, in particular the LSS, where there is a regular program of ophthalmological examinations^19^. Because the radiologic technologists in this cohort were medically literate, one would expect that self-reports of ocular and other medical outcomes, such as diabetes, would be reasonably reliable. This is supported by the fact that, when medical records were obtained, 89% of cancers reported on the second survey were accurately reported^39^. However, it is possible that technologists with glaucoma and macular degeneration would be likely to recall and report the well-known risk factors for these endpoints than those without. Another weakness is that, as with many occupational studies, cohort members had to survive to answer the second questionnaire. However, this will not necessarily bias our analysis because everyone had to survive to answer a questionnaire, and all risk was assessed conditional on that.

In general, the non-significant risks we observed for glaucoma and macular degeneration were statistically consistent with those observed in other radiation-exposed groups, as shown in Tables 3 and 4. In particular, the trend with dose for glaucoma in the Japanese atomic-bomb survivors had a borderline significant (*p* = 0.025) negative slope^40^, which is consistent with the (statistically non-significant) trend observed in the radiological technologists (Table 3). However, when additional adjustment was made for distal-proximal status (a surrogate for urban-rural status) in the atomic bomb survivors, the statistical significance was much weakened (*p* = 0.14), although the central trend estimate was essentially unchanged^40^. Minamoto *et al*. reported a (non-significant) positive trend for intraocular pressure with radiation dose in the Japanese atomic-bomb survivors^20^. Kiuchi *et al*.^19^ reported a significant radiation-associated excess risk for normal-tension glaucoma, although not for other types of glaucoma. There was a significant positive trend with dose for retinal degeneration in the Japanese atomic-bomb survivors^20^, although there was no significant dose trend in the more etiologically relevant study by Itakura *et al*.^21^. Both trends are consistent with the (non-significant) positive trend observed in the radiological technologists (Tables 3 and 4).

**Table 4:** Risks for glaucoma and macular degeneration in US radiologic technologists and other radiation-exposed cohorts.

Model	Dose (Gy), mean (range)	Cases	Excess relative risk (hazard ratio) /Gy	95% CI
**Glaucoma**				
US radiologic technologists (current study, unadjusted for covariates)	0.058 (0–1.51)	1631	−1.39	(−1.94, −0.69)
US radiologic technologists (current study, adjusted for covariates)			−0.57	(−1.46, 0.60)
Japanese atomic-bomb survivors^40^	0.57 (0 − >4.14)	211	−0.18	(−0.20, −0.03)
Japanese atomic-bomb survivors^19^ – primary open-angle normal tension glaucoma (IOP ≤21 mmHg)	0.47 (0 − >3.01)	226	0.31	(0.11, 0.53)
Japanese atomic-bomb survivors^19^ – primary open-angle hypertensive glaucoma (IOP >21 mmHg)		36	−0.21	(−0.48, 0.21)
Japanese atomic-bomb survivors^19^ – primary angle-closure glaucoma		25	−0.46	(−0.71, 0.02)
**Macular degeneration**				
US radiologic technologists (current study, unadjusted for covariates)	0.058 (0−1.51)	1331	0.73	(−0.01, 1.74)
US radiologic technologists (current study, adjusted for covariates)			0.32	(−0.32, 1.27)
Japanese atomic-bomb survivors: retinal degeneration^20^	NA (0 − >3.0)	55	0.42	(0.07, 0.88)
Japanese atomic-bomb survivors early age-related macular degeneration^21^	0.45 (0 − >2.0)	191	−0.07	(−0.25, 0.15)
Japanese atomic-bomb survivors late age-related macular degeneration^21^		6	−0.21	(−0.79, 1.94)

Adjusted relative risks for the present study are evaluated using a Cox model with age as timescale, with stratification by sex, race and birth year (by decade <1900, 1900–1909, …, 1950–1959, ≥1960), and with adjustment to the baseline hazard for diabetes, body mass index (BMI), smoking status (current smoker/ex-smoker/never smoker, numbers of cigarettes/day, age stopped smoking), each ascertained at the baseline survey. For each endpoint (glaucoma, macular degeneration) follow-up is restricted to those persons eligible for study (no record of radiotherapy or disease at baseline, unambiguous status at end of follow-up (with both fact and year of diagnosis known) etc)(see Methods). IOP = intraocular pressure.

In conclusion, the present large occupational study of low-dose and low dose-rate radiation exposure finds little evidence of radiation-associated excess risks for glaucoma or macular degeneration. The absence of glaucoma risk following the low doses received contrasts with the well-documented risks for neovascular glaucoma at high doses, and the single report of normal-tension glaucoma at lower doses^19^, suggesting that glaucoma in aggregate may be a type of tissue reaction effect^32^, although with differing contributions from the various subtypes at differing levels of dose. The difference between the unadjusted and adjusted analyses highlights the importance of collecting relevant covariate lifestyle and environmental risk factor data in studies of these endpoints. Ours is the first study of a group exposed at low doses and low dose rates^22^. It will be important to continue follow-up in this cohort and, if possible, to clinically validate and characterise future ocular lesions. It is highly desirable that glaucoma and macular degeneration risks associated with low doses are assessed in other radiation-exposed groups with well-validated dosimetry, medically-confirmed ophthalmological examination, and data on relevant lifestyle and environmental risk factors.

## Methods

### Study population and follow-up

The USRT study population and methods have been described elsewhere^23,41^. Briefly, the US National Cancer Institute, in collaboration with the University of Minnesota and the American Registry of Radiologic Technologists (ARRT), is studying cancer incidence and mortality among 146,022 (106,953 women) US radiologic technologists who were certified for at least two years during 1926–1982^41^. Active follow-up was conducted through yearly re-certification with the ARRT. Inactive registrants were linked with national and other databases, including the Social Security Administration and National Death Index, to determine vital status and obtain causes of death. Four different questionnaires were administered during the periods 1983–1989, 1994–1998, 2003–2005, and 2012–2014 to collect information on health outcomes (including self-reports of glaucoma and macular degeneration, except in the first questionnaire), work history, demographic and lifestyle characteristics, and medical histories. The response rate for each of the questionnaires among living and located cohort members was approximately 68–72% for the first three surveys and 63% for the last survey. A total of 110,373 radiological technologists completed at least one of the first two questionnaires and are considered study participants.

Data on glaucoma, and macular degeneration were only elicited on the second through fourth questionnaires. Based on responses to the first two questionnaires we excluded persons who indicated that they had received radiotherapy. We also censored follow-up after a diagnosis of cancer other than non-melanoma skin cancer (NMSC) because of the potential for radiotherapy that the subject might receive, resulting in a reduction of 87,827/88,594 person years for glaucoma/macular degeneration, respectively. After excluding 8252 technologists who reported a history of radiotherapy on the first or second surveys, and (a) a further 737 with inconsistent questionnaire responses for glaucoma and 31,816 who were not informative for glaucoma [because they had glaucoma at baseline, or gave incomplete information in subsequent questionnaires], or (b) 567 with inconsistent responses for macular degeneration and a further 31,585 not informative for this endpoint [for analogous reasons as those for glaucoma], there were a total of 69,568/69,969 technologists eligible for study of glaucoma/ macular degeneration, respectively. Further details on eligibility criteria are given in Supplementary Information Part A.

Individuals were deemed at risk for developing glaucoma or macular degeneration during any of the combinations of time between completion of the (a) second-to-third questionnaires, (b) second-to-fourth questionnaires, and (c) third-to-fourth questionnaires. For each endpoint individuals were included in each inter-questionnaire period during which they were initially free of disease (i.e., did not report the endpoint at the first of the pair of questionnaires), and had unambiguous indication of disease diagnosis (and year of diagnosis) at the second of the pair of questionnaires. Follow-up terminated for each endpoint at the earliest of (a) date of first diagnosis of the respective endpoint, (b) date of diagnosis of any cancer other than NMSC, or (c) completion-date of final questionnaire. The consistency criteria (Supplementary Information Part A) applied meant that the union of all eligible intervals could be employed for follow-up.

### Dosimetry

A historical dose reconstruction was undertaken to estimate annual radiation absorbed doses to specific organs from occupational exposure for each radiologic technologist, described in more detail in Supplementary Information Part B and in Simon *et al*.^23^. Annual reported badge doses were used for each radiological technologist when available; otherwise, doses were estimated from probability density functions based on population exposure data for each year worked, modified by a work history questionnaire-derived exposure score. All annual reported badge doses, in terms of personal dose equivalent (mSv), were estimated up to December 31^st^ 1997. Questionnaire response was used to modify badge doses for the estimation of eye-lens absorbed doses. The doses reflect exposures from performing or assisting in diagnostic and therapeutic radiological procedures, and were mostly from x-rays^23^. Most radiologic technologists performed or assisted with multiple procedures, including standard fluoroscopy, multi-film and routine diagnostic radiography, and some worked with fluoroscopically-guided interventional or radio-pharmaceutical procedures^23^. Eye-lens absorbed doses were estimated from measured or estimated personnel monitoring badge doses, badge-dose-to-organ-absorbed-dose conversion factors based on beam energy (kV) and x-ray beam filtration specific to each kV and time period^23^.

### Covariates

The following variables were selected *a priori* as adjusting covariates in most regressions because of their known effect on glaucoma or macular degeneration prevalence^8–10^, and because they were regarded as potentially confounding the relationship with radiation dose, namely sex, racial/ethnic group, birth year, diabetes, body mass index (BMI), smoking status (current smoker/ex-smoker/never smoker, numbers of cigarettes/day, age stopped smoking), each ascertained at the baseline survey. For both endpoints diabetes is likely to result in increased frequency of eye exam, with consequent increased likelihood of detection of the disease. As such, we judged it important to adjust for diabetes status, irrespective of its status as a risk factor. Because of the associations of radiotherapy with glaucoma and the potential for missing dose, we also excluded any persons with history of such treatment recorded on either of the first two questionnaires for both endpoints. We also censored follow-up at the point of development of any cancer other than NMSC. Nevertheless, we also show analysis in which persons with radiotherapy were added back in or cancer censoring was removed (Supplementary Information Part C Table 1). Preliminary analyses suggested that ultraviolet radiation (UVR) had only a very slight effect on glaucoma or macular degeneration risk. Since it was therefore unlikely to confound the radiation dose response, models did not adjust for UVR. When not available from the second questionnaire, data for the *a priori*-specified potential confounding variables were obtained from first questionnaire for those who completed both questionnaires (details given below).

### Statistical methods

Risks for glaucoma and macular degeneration were assessed using Cox proportional hazards models^42^, with age as the timescale, in which the relative risk (hazard ratio) for individual $i$ at age $a$ was given by:

1 $$R{R}_{i}[a,{D}_{i},({X}_{ji})|\alpha ,({\beta }_{j})]=\exp [\sum _{j=1}^{N}{\beta }_{j}{X}_{ji}][1+\alpha {D}_{i}(a-5)]$$

where ${D}_{i}(a-5)$ is the time-varying cumulative eye-lens absorbed dose in Gy from occupational exposures, lagged by 5 years to allow for disease latency^43^, $({X}_{ji})$ are the lifestyle/medical risk factors, $\alpha$ is the excess relative risk coefficient (ERR) per unit dose (Gy), and $({\beta }_{j})$ are coefficients adjusting for lifestyle risk factors. The relative risks (hazard ratios) given in Tables 1 and 2 were derived using this model without any additional adjustment. In Table 2, the dose used is the cumulative eye-lens absorbed dose from occupational exposures up to December 31, 1997, the final date to which these doses were estimated. In Table 3 and Fig. 1, eye-lens absorbed dose was treated as a time varying measure, and lagged by 5 years.

Analyses of all endpoints in Table 3 and Fig. 1 were adjusted by stratification for sex, race, and year of birth (by decade), and via adjustment to the baseline hazard for the *a priori*-determined risk factors for glaucoma and macular degeneration, as given above. All analyses were carried out using R^44^ and Epicure^45^. All tests were 2-sided.

### Ethical approval

Informed consent was obtained from all participants. The study was approved by the National Cancer Institute Special Studies Institution Review Board and by the University of Minnesota Institution Review Board. All methods were performed in accordance with the relevant guidelines and regulations of the National Institutes of Health.

### Data availability statement

All data used for the analysis is available from the principal author on request.

## Electronic supplementary material

Supplementary Information: Occupational radiation exposure and glaucoma and macular degeneration in the US radiologic technologists. Supplementary Information Parts A, B, C
