# Supplementary material for: Occupational radiation exposure and glaucoma and macular degeneration in the US radiologic technologists
Source: Sci Rep. 2018 Jul 11;8:10481. doi: 10.1038/s41598-018-28620-6 (PMC6041262; doi:10.1038/s41598-018-28620-6)
Supplement: Supplementary file 1 — Supplementary Information: Occupational radiation exposure and glaucoma and macular degeneration in the US radiologic technologists. Supplementary Information Parts A, B, C [file 41598_2018_28620_MOESM1_ESM.pdf]

Supplementary Information

Occupational radiation exposure and glaucoma and macular degeneration in the US radiologic technologists

Mark P. Little, Cari M. Kitahara, Elizabeth K. Cahoon, Marie-Odile Bernier, Raquel Velazquez-Kronen, Michele M. Doody, David Borrego, Jeremy S. Miller, Bruce H. Alexander, Steven L. Simon, Dale L. Preston, Craig Meyer, Martha S. Linet, Nobuyuki Hamada

Supplementary Information Part A. Algorithm for determining whether a person is in follow-up for glaucoma or macular degeneration, and dates of beginning and end of follow-up

We outline the algorithm for determining eligibility of each subject to be in the glaucoma analysis. With obvious changes to certain variables the same algorithm is used to determine eligibility of a person for analysis of macular degeneration. Supplementary Information Part A Table 1 gives the numbers of persons in the analysis cohort resulting from successive exclusions (step 6). Q1–Q4 correspond to the first to fourth questionnaires.

1. Calculate a logical variable recording whether any radiotherapy (RT) was recorded on Q1 or Q2:

   RT_Q1_Q2 = IF([ever had therapeutic X ray on Q1] OR [ever had RT on Q2])

2. Calculate a logical variable recording whether the glaucoma data on Q2/Q3/Q4 questionnaires is consistent, in that if glaucoma is recorded on an earlier questionnaire then it is recorded on all later questionnaires:

   GLAUCOMA_CONSISTENCY

   = NOT[{(answered Q2) & (answered Q3) & (diagnosed with glaucoma at Q2) & (not diagnosed with Glaucoma at Q3)} OR

   {(answered Q2) & (answered Q4) & (diagnosed with glaucoma at Q2) & (not diagnosed with glaucoma at Q4)} OR

   {(answered Q3) & (answered Q4) & (diagnosed with glaucoma at Q3) & (not diagnosed with glaucoma at Q4)}]

3. Calculate a logical variable recording whether the Q2/Q3 questionnaire data is informative for glaucoma, including its timing (they must not have glaucoma at Q2, and not be in the situation at Q3 where it is known that they had glaucoma but the year in which they had it is unknown):

GLAUCOMA_Q2_Q3_OK

= (answered Q2) & (answered Q3) & (not diagnosed with glaucoma at Q2) & NOT{(diagnosed with glaucoma at Q3) & [year diagnosed with glaucoma at Q3 is missing/non-numeric)}

4. Calculate a logical variable recording whether the Q2/Q4 questionnaire data is informative for glaucoma, including its timing (they must not have glaucoma at Q2, and not be in the situation at Q4 where it is known that they had glaucoma but the age at which they had it is unknown):

   GLAUCOMA_Q2_Q4_OK

   = (answered Q2) & (answered Q4) & (not diagnosed with glaucoma at Q2) & NOT{(diagnosed with glaucoma at Q4) & [age diagnosed with glaucoma at Q4 is missing/non-numeric)}

5. Calculate a logical variable recording whether the Q3/Q4 questionnaire data is informative for glaucoma, including its timing (they must not have glaucoma at Q3, and not be in the situation at Q4 where it is known that they had glaucoma but the age at which they had it is unknown):

   GLAUCOMA_Q3_Q4_OK

   = (answered Q3) & (answered Q4) & (not diagnosed with glaucoma at Q3) & NOT{(diagnosed with glaucoma at Q4) & [age diagnosed with glaucoma at Q4 is missing/non-numeric)}

6. Determine eligibility of subject for analysis of glaucoma via the logical variable:

   GLAUCOMA_ELIGIBLE

   = NOT(**RT_Q1_Q2**) & (**GLAUCOMA_CONSISTENCY**) &

   [**GLAUCOMA_Q2_Q3_OK** OR **GLAUCOMA_Q2_Q4_OK** OR

   GLAUCOMA_Q3_Q4_OK]

7. Calculate a date variable recording date of start of follow-up:

   DATE_START =

```
IF (answered Q2) THEN
  = Q2 response date
ELSE
   IF (answered Q3) THEN
      = Q3 response date
   ELSE
      = Q1 response date
   END IF
END IF
```

8. Calculate a date variable recording date of final questionnaire answered:

   DATE_FINAL_QUEST =

```
MAX{
  IF (answered Q1) THEN
      = Q1 response date
  ELSE
      = birth date
  END IF,
  IF (answered Q2) THEN
    = Q2 response date
  ELSE
      = birth date
  END IF,
  IF (answered Q3) THEN
```

= Q3 response date

ELSE

= birth date

END IF,

IF (answered Q4) THEN

= Q4 response date

ELSE

= birth date

END IF}

9. Calculate date at end of follow-up, taking account of first incident cancer (apart from NMSC) via the date variable:

   DATE_END_CANCER =

   IF (invasive or in situ cancers excluding NMSC occurs) THEN

   =MIN[**DATE_FINAL_QUEST**, cancer diagnosis date]

   ELSE

   = **DATE_FINAL_QUEST**

   END IF

10. Calculate date for end of follow-up of glaucoma [last informative answered questionnaire], ignoring possible cancer incidence (apart from NMSC), via the date variable:

    DATE_QU_END_GLAUCOMA_NO_CAN =

    IF (**GLAUCOMA_Q2_Q4_OK** OR **GLAUCOMA_Q3_Q4_OK**) THEN

    = Q4 response date

    ELSE IF (**GLAUCOMA_Q2_Q3_OK**) THEN

    = Q3 response date

ELSE

= **DATE_FINAL_QUEST**

END IF

11. Calculate date for end of follow-up of glaucoma [taking account of glaucoma diagnosis age/year], ignoring possible cancer incidence (apart from NMSC) via the date variable:

    DATE_END_GLAUCOMA_NO_CAN =

    MIN{

    IF [diagnosed with glaucoma at Q3] THEN

    IF [year diagnosed with glaucoma at Q3 is missing/non-numeric] THEN

    = **DATE_QU_END_GLAUCOMA_NO_CAN**

    ELSE

    = [year diagnosed with glaucoma at Q3] / 7 / 2 [=mid year estimate of glaucoma]

    END IF

    ELSE

    = **DATE_QU_END_GLAUCOMA_NO_CAN**

    END IF,

    IF [diagnosed with glaucoma at Q4] THEN

    IF [age diagnosed with glaucoma at Q4 is missing/non-numeric] THEN

    = **DATE_QU_END_GLAUCOMA_NO_CAN**

    ELSE

    = birth date + [age diagnosed with glaucoma at Q4+0.5] [=mid birth-year estimate of glaucoma]

    END IF

    ELSE

    = **DATE_QU_END_GLAUCOMA_NO_CAN**

END IF}

12. Calculate date for end of follow-up of glaucoma, taking account of glaucoma diagnosis age/year and taking account of possible cancer incidence (apart from NMSC) via the date variable:

    DATE_END_GLAUCOMA_CAN

    =MIN{ DATE_END_GLAUCOMA_NO_CAN, DATE_END_CANCER}

Supplementary Information Part A Table 1. Numbers of persons in the analysis cohort resulting from successive exclusions

	Endpoint	
Successive exclusions	Glaucoma	Macular degeneration
Original data	110,373	110,373
No radiotherapy (Q1+Q2)	102,121	102,121
Above + consistency for endpoint	101,384	101,554
Above + informative for endpoint Q2+Q3 or Q2+Q4 or Q3+Q4	69,568	69,969

Supplementary Information Part B Summary of estimation methods for occupational radiation dose to the lens of the eye

The individual dose estimates to the lens of the eye used in these analyses were obtained from the current USRT dosimetry system described in detail in Simon *et al* [1]. This supplement provides a summary of the methods for reconstructing individual annual eye lens doses used in these analyses.

Efforts to reconstruct individual annual occupational doses for members of the USRT were first described in Simon *et al* [1] with later improvements discussed in detail in Simon [2] and Simon *et al* [1]. The basic strategy for estimation of absorbed dose to the lens of the eye is to convert an estimate or measurement of the exposure from a personnel monitoring device (e.g., a film badge) to the air kerma that produced that measurement, and to then convert the air kerma to the eye-lens absorbed dose for a worker exposed to that level of air kerma. [*Air kerma* is an acronym for "kinetic energy released per unit mass of air", defined as the sum of the kinetic energies of all the charged particles (e.g. electrons) liberated by the x-rays that ionise the air molecules (divided by the mass of the air).] Typically, personally monitoring devices reflect weekly, bi-weekly, or monthly exposure to ionising radiation. Historical units of individual exposure and dose have changed over time. For consistency, all estimates or reported measurements from personal monitoring measurements, regardless of the working years for individual technologists, have been converted to mSv (milli-sievert), the present international standard for the personal dose equivalent, and all eye lens absorbed dose are reported in units of mGy (milligray). [*Personal dose equivalent* is defined by the International Commission on Radiation Units and Quantities[3].]

Several assumptions are necessary to complete the calculations described above, in particular, the energy distribution of the x-ray field, the spatial homogeneity of the radiation field in the environment where each technologist worked, and the geometry of the irradiation.

Data used to characterise these assumptions were derived from common practice and numerous research findings as discussed in Simon [2].

The number of cohort members for which dose estimates were necessary and the volume of personnel monitoring data for the group varied by time-period. About 1,000,000 annual eye doses were estimated for the 90,305 cohort members who responded to the first USRT mail survey. These estimates were based on 350,000 individual annual badge dose readings for years between 1960 and 1984, while for the period before 1960, individual doses were derived from annual population dose distributions obtained from the historical literature for those years, supplemented with work history information from the first survey.

The current dosimetry system provides about 2.2 million estimates of annual eye lens dose for years through 1997 for the 110,373 cohort members who responded to at least one of the first two mail surveys. These estimates were based on more than 900,000 individual annual badge dose readings for the period from 1960 through 1997 with most of the badge dose readings obtained from 1976 onward.

Numerous considerations were necessary to properly convert from reported or measured badge doses including, accounting for (i) measurement error in individual film-badge readings; (ii) badge doses below the minimum detection limit; (iii) attenuation of the readings when badges were worn underneath a protective lead apron; and (iv) for the number of working hours and variations in practices in years for which an individual badge dose reading was unavailable. Considerable discussion on these issues is provided in Simon *et al* [1].

Uncertainty of estimated doses was an important consideration in the design of the dosimetry system. Dose uncertainty was quantitatively estimated by first considering the uncertainty of the individual parameters used in the dose calculations and to characterise each by a probability density function. The general method was to then use Monte Carlo methods to propagate the individual parameter uncertainties and to derive the uncertainty on the eye lens

dose. Because some of these parameters, such as uncertainties in the literature-based population mean values for early years, were shared between individuals and others varied from person-to-person or even year-to-year within a person, a method for generating multiple sets of alternative doses for the cohort [4] was used. Each set of annual doses for all cohort members computed with a common set of shared parameters is termed a dose realisation. The presence of shared uncertainties results in correlations between individuals across realisations.

For these analyses, 1,000 realisations of individual annual air kerma and eye lens dose estimates were produced. The 1,000 realisations for a given year for an individual cohort member can be thought of as a sample from the distribution of the individual's true dose for that year given what is known about their work history and practices together with the uncertainties in the population distributions. The individual annual dose estimates used in analyses were regression-calibration estimates computed as the means over the 1,000 Monte Carlo realisations.

Supplementary Information Part C. Supplementary tables

Supplementary Information Part C Table 1. Sensitivity analysis when persons with radiotherapy recorded on either of first two questionnaires, or persons with cancer are not censored. Other notes are as for Table 3 in the main text, for fully adjusted analysis.

	Glaucoma			Macular degeneration		
	Cases	ERR / Gy (+95% CI)	*p*-value	Cases	ERR / Gy (+95% CI)	*p*-value
Q1+Q2 persons with radiotherapy not excluded	1769	-0.41 (-1.23[w], 0.71)	0.428	1474	0.18 (-0.38, 1.00)	0.579
Persons with cancer not censored	1631	-0.79 (-1.51, 0.31)	0.141	1331	0.17 (-0.40, 1.02)	0.623
Q1+Q2 persons with radiotherapy not excluded, persons with cancer not censored	1769	-0.56 (-1.33, 0.53)	0.278	1474	0.10 (-0.42, 0.87)	0.755

References

1 Simon, S. L. *et al.* Radiation organ doses received in a nationwide cohort of U.S. radiologic technologists: methods and findings. *Radiat. Res.* **182**, 507-528, doi:10.1667/RR13542.1 (2014).

2 Simon, S. L. Organ-specific external dose coefficients and protective apron transmission factors for historical dose reconstruction for medical personnel. *Health Phys.* **101**, 13-27, doi:10.1097/HP.0b013e318204a60a [doi];00004032-201107000-00002 [pii] (2011).

3 Allisy, A., Jennings, W. A., Kellerer, A. M. & Müller, J. W. ICRU Report 51. Quantities and units in radiation protection dosimetry. *J. Int. Commission Radiat. Units Meas.* **os26**, NP-NP, doi:10.1093/jicru/os26.2.Report51 (1993).

4 Simon, S. L., Hoffman, F. O. & Hofer, E. The two-dimensional Monte Carlo: a new methodologic paradigm for dose reconstruction for epidemiological studies. *Radiat. Res.* **183**, 27-41, doi:10.1667/RR13729.1 (2015).
